# Williams Syndrome Associated With Autism Spectrum Disorder: A Case Report and Review of the Literature

**DOI:** 10.7759/cureus.9641

**Published:** 2020-08-10

**Authors:** Max M Carvalho, Jeanne Mazza

**Affiliations:** 1 Faculty of Medicine, University of Brasília, Brasília, BRA; 2 Pediatric Neurology and Neurogenetics, Brasília University Hospital, Brasília, BRA

**Keywords:** william's syndrome, asd, autism spectrum disorder, neurology, pediatrics, pediatric neurology, genetics

## Abstract

We present a case report of patient H., a four-year-old male who was brought to the neurogenetics clinic of a university hospital. He was diagnosed with Williams syndrome (WS), which was confirmed by microarray; the patient was also diagnosed with autism spectrum disorder (ASD) by the pediatric neurology department. It is known that due to WS's phenotypical pattern, a co-diagnosis of both ASD and WS is apparently unlikely and often ignored. This case report discusses how this matter is addressed in the literature to understand the overlap of these two diseases, treating this case as a study model.

## Introduction

Williams-Beuren syndrome, or simply Williams syndrome (WS), is a rare chromosomal abnormality with an incidence rate of one out of 20,000 live births [1]. WS is mainly caused by 7q11.23 microdeletion, usually a "de novo" mutation. Important genes that are involved in the syndrome physiopathology include ELN, coding for Elastin. Its underexpression may result in the development of supravalvar aortic stenosis, which is commonly found in WS patients [2]. This chromosomal aberration has systemic effects, leading to global developmental delay, and causing several symptoms and signs. A flattened nasal bridge, shorter nose, enlarged frontal region, thinner chin, thicker lips, larger mouth, and periorbital fullness are the craniofacial morphology alterations usually identified in WS patients [1,2]. Regarding neurocognitive and neuropsychomotor development, children with WS show lower than average IQ, ranging between 60-70, thus constituting mild to moderate intellectual disability. They also often have limb hypotonia and consequent motor development general delay. Finally, significant difficulties regarding visual and spatial cognition have also been reported in such children. However, facial processing and language have been shown by many studies to be preserved in this population, even though this finding happens to be a controversial topic in the literature. A mostly universal fact regarding WS patients is their strong interest in music, despite not being able to deal well with noises of high intensity [1-3].

Autism spectrum disorder (ASD) is a neurodevelopmental disorder characterized by two main signs: multiple deficits in social interactions and communication, and repetitive or stereotyped behaviors. It is accepted today that both genetic and environmental factors may lead to the development of ASD in a patient [4].

## Case presentation

We discuss the case of H., a four-year-old African-American boy, who was referred to the neuropediatrics department of a university hospital by the genetics department. He was admitted with a WS diagnosis through microsomal analysis by microarray. His mother reported developmental delay in the child, along with gait and language difficulties. H. had been born by cesarean delivery due to alterations detected on ultrasound and restricted growth. Some interesting factors observed during the first appointment were as follows: the infant was just starting to learn how to speak, and was showing an evident delay in speech development; he did not have sphincter control and was highly irritable. Regarding social interactions, he was able to make conversations with other people, but very poorly. He would also often hit his head on the floor, and slap his own face when annoyed. H. could not eat by himself either, as he had difficulty in chewing. H. had been born when his mother was 34 years old, and she already had another child at the time. She and H.'s brother were both healthy, but the patient's father had bipolar disorder. The mother also claimed that H.'s paternal family has many members with psychiatric and behavioral disorders.

During the consultation, the patient showed mood swings. At first, he was euphoric and appeared somewhat interested in new people and objects, with greater attention shown on the latter. At other times, he was found to be very irritable and not quite communicative, exhibiting the self-injurious behaviors already described, and was being disruptive to the point of not allowing physical examination. Only playing certain songs would calm him down. The patient had only recently had early left stimulation, acquiring great and remarkable development in language, with an outstanding expansion of his vocabulary; he had become more articulate and started to form sentences with more than three words. However, his language was limited to being more instrumental, meaning that he would rather talk for more pragmatic and immediate purposes, such as asking for a glass of water. Some of his utterances were still incomprehensible and had some vocal stereotypes. He had started to attend school and daycare but had shown minimal intimacy toward schoolmates or teachers, albeit showing no conflict while being with them. Regarding daily living activities, he had an important deficiency. His most frequent stereotype was rotating on his own axis. He also initially had the habit of tiptoeing but had abandoned it. In general, he showed an irregular social smile and preferred to spend his time with inanimate objects for the most part, mostly showing no interest in strangers at the clinic with a few exceptions, as described earlier. At the time of the physical examination, he showed extreme resistance to being touched and initiated self-injurious behavior.

As for neuropsychomotor development, H. had started to sit without support at eight months and had started to pick up objects at the age of one year; he had started to walk only by one year and seven months. By the time he reached his current age of four, he started pointing to things he wanted or showed interest in; but he still did not make any gestures, and relied on the instrumental use of adults when he wanted something. His gait was rather ataxic, with static and dynamic imbalance, in addition to the presence of hypotonia in the upper and lower limbs.

Regarding complementary exams, the electroencephalogram was normal for his age. The audiometry, on the other hand, was impaired by the patient's refusal to put on earphones; hence, the exam was performed in a free field, but no significant hearing loss was detected initially. Finally, due to his continuing disruptive behavior, levomepromazine 1% was prescribed.

## Discussion

The joint occurrence - and diagnosis - of WS and ASD is still a very controversial subject. Many authors commonly describe the two disorders as being opposites to each other [5]. This is due to the behavioral and social profile of children with WS being that of very outgoing, friendly, and affable individuals, having no difficulty in initiating interactions with other people, even to the point of easily embracing strangers [2]. However, there is evidence that patients fluent in speech with WS carry some symptoms associated with ASD, with an average frequency of 1:3 for every case, especially related to verbal, communicative, and language development [6,7]. Despite this, children who are learning how to speak appear to have greater difficulty, indicating that a diagnosis of ASD may need some patience in view of its development.

To the best of our knowledge, there are five other case reports, or case series, in the literature that portray the joint occurrence of WS and ASD [8-12]. However, some of these reports, such as the one published in Sweden by Gillberg and Rasmussen, were prepared when it was not even known that WS was caused by a microdeletion on chromosome 7. A larger, more recent, and more in-depth study by Tordjman et al., which used the Autistic Diagnostic Observation Schedule (ADOS) methodology, has found the presence of symptoms in nine French individuals with a WS diagnosis confirmed by fluorescent in situ hybridization (FISH) [13]. Bearing in mind the limits of our knowledge regarding the topic, this is the first case report of a patient diagnosed with ASD and 7q11.23 microdeletion in Brazil.

In general, the patient clearly did not present the behavioral and social profile associated with WS in a marked or well-defined manner. On the other hand, the ASD diagnosis is not so simple, especially if a look is taken at the first part of the described encounter with the patient; but the patient's behavior at home as reported by the mother along with the observations made during the second part of the mentioned consultation justified the diagnosis of ASD level 1 or 2 according to the Diagnostic and Statistical Manual of Mental Disorders, 5th Edition (DSM-V) criteria, in addition to an intellectual disability that is yet to be specified.

Contrary to what is commonly perceived about WS, everything we know about the condition indicates that these children appear to have considerable difficulties with regard to adequate social development. Interestingly, one of the social subtypes proposed to be associated with ASD, the active albeit strange one, has remarkable similarities with the WS behavioral phenotype [14]. More often than not, these individuals seek social interaction, even with strangers, and notably more intensely than their neurotypical peers or peers with other developmental problems [6]. However, they find it difficult to continuously talk about the same topic in a discussion, especially if their interlocutor has chosen the subject, and they also show issues with other finer social skills, such as perceiving subtle forms of humor. Another point worthwhile to note is that these children are able to have some form of comprehension of other individuals' feelings, but do not use this information properly, or even correspond to it. Patient H. demonstrated this phenomenon: when his mother tried to signal that she wanted a hug, he approached her ready to receive it, but did not return his mother's embrace; this indicated that the patient lacked the element of emotional reciprocity and, therefore, effective communication. It is evident that this willingness of the patient with WS to establish contact can cause quite some confusion in terms of effectively acknowledging their actual social-relation difficulties.

It is also worthwhile to note that the symptoms, that of WS's in particular, do not appear to be related to developmental delay alone, being therefore linked to another pathology. ASD symptoms would not be, as such, contained inside the WS phenotype; after all, many children diagnosed with WS do not show the mentioned difficulties as described [6,15].

## Conclusions

The diagnosis of ASD is solely and exclusively dependent on the presentation or lack of the behavioral criteria that characterize it. The presence of any other comorbidity should in no way cause the correct diagnosis to be disregarded. Even though most patients with WS may not have enough symptoms for a diagnosis of ASD, characteristic signs must be noticed so that these children can have access to evidence-based interventions that meet their needs. The socially active pattern and the interest in establishing contact with others shown by the patient must never preclude a prompt analysis of these signs by the clinician. It is also proposed that WS encompasses a variable spectrum of behavioral manifestations, a possibility that should be studied further for a better phenotypic understanding of this syndrome.
